# DNA Damage in Healthy Individuals and Respiratory Patients after Treating Whole Blood *In vitro* with the Bulk and Nano Forms of NSAIDs

**DOI:** 10.3389/fmolb.2016.00050

**Published:** 2016-09-28

**Authors:** Mojgan Najafzadeh, Charmaine Normington, Badie K. Jacob, Mohammad Isreb, Rajendran C. Gopalan, Diana Anderson

**Affiliations:** ^1^Division of Medical Sciences, School of Life Sciences, University of BradfordBradford, UK; ^2^Bradford Royal InfirmaryBradford, UK; ^3^St Luke's HospitalBradford, UK; ^4^School of Pharmacy, University of BradfordBradford, UK

**Keywords:** DNA damage, lung cancer, COPD, asthma, nanoparticles, bulk forms aspirin, ibuprofen

## Abstract

Non-steroidal anti-inflammatory drugs (NSAIDs) inhibit COX enzyme activity which affects the inflammatory response. Inflammation is associated with increasing cancer incidence. Pre-clinical and clinical studies have shown that NSAID treatment could cause an anti-tumor effect in cancers. In the present study, blood was taken from healthy individuals (*n* = 17) and patients with respiratory diseases or lung cancer (*n* = 36). White blood cells (WBC) were treated with either a micro-suspension, i.e., bulk (B) or nano-suspension (N) of aspirin (ASP) or ibuprofen (IBU) up to 500 μg/ml in the comet assay and up to 125 μg/ml in the micronucleus assay. In this study results were compared against untreated lymphocytes and their corresponding treated groups. The results showed, that NSAIDs in their nano form significantly reduced the DNA damage in WBCs from lung cancer patients in bulk and nano compared to untreated lymphocytes. Also, there was a decrease in the level of DNA damage in the comet assay after treating WBCs from healthy individuals, asthma and COPD groups with aspirin N (ASP N) but not with IBU N. In addition, the number of micronuclei decreased after treatment with NSAIDs in their nano form (ASP N and IBU N) in the healthy as well as in the lung cancer group. However, this was not the case for micronucleus frequency in asthma and COPD patients. These data show that lymphocytes from different groups respond differently to treatment with ASP and IBU as measured by comet assay and micronucleus assay, and that the size of the suspended particles of the drugs affects responses.

## Introduction

Extensive pre-clinical data and a limited number of clinical investigations have proposed a direct effect of non-steroidal anti-inflammatory drugs (NSAIDs) on tumor biology, with an anti-tumor effect on several of the hallmarks of cancer, including proliferative capacity, evasion of apoptosis and cell cycle regulation, and invasive capability of tumor cells (Park et al., 2014). Furthermore, clinical evidence has suggested a pertinent role in down-regulating the systemic inflammatory response whilst favorably influencing the local inflammatory response within the tumor microenvironment (Hussain et al., 2012). Despite such compelling results, the clinical applicability of NSAIDs, statins and histamine-2 receptor antagonists has not been fully realized, particularly in patients identified at high risk on the basis of inflammatory parameters (Pasche, 2013). Most of the studies with NSAIDs have been conducted with gastrointestinal cancers (colorectal, gastric, esophagus), etc. A study examined the potential role that these agents may play in improving survival and reducing recurrence in patients with potentially curative colorectal cancer, with a particular focus on their effects at the local and systemic inflammatory response (Mansouri et al., 2013).

A clinical trial study examined the association between developing cancer and long-term, taking of daily low-dose aspirin (100 mg) and placebo in healthy women (Cook et al., 2013). There was no extended effect on cancer deaths or colorectal polyps, however, the side effects of aspirin such as gastrointestinal bleeding and peptic ulcers were statistically noticed (Cook et al., 2013). With few exceptions, however, epidemiological studies have found that individuals who take NSAIDs have a reduced risk of colorectal adenomas and carcinoma (Baron, 2009). Similarly, randomized studies in patients with familial adenomatous polyposis have uniformly found that NSAIDs can lead to polyp regression and prevention of new polyps, and trials in patients with sporadic adenomas document that aspirin reduces the risk of adenoma recurrence. Burn et al. (2011) examined the effect of aspirin on cancer risk in carriers of hereditary colorectal cancer in the Colorectal Adenoma/Carcinoma Prevention Programme (CAPP) randomized controlled trial. They concluded that 600 mg per day for a mean of 25 months reduced cancer incidence after 58 months in carriers of hereditary colon cancer (Burn et al., 2011). They are planning studies to establish the optimum dose and duration of treatment (Burn et al., 2011, 2013). Together these data provide convincing evidence for the chemopreventive efficacy of NSAIDs in the large bowel (Baron, 2009; Brady et al., 2011; Seufert et al., 2013).

There is more evidence that shows a chemopreventive effect for aspirin and other NSAIDs on colorectal cancer (Coghill et al., 2012) and other cancer types such as prostate cancer (Kawahara et al., 2010), oesophageal (Kang et al., 2013), gastric (Tian et al., 2010), and breast (Brasky et al., 2011; Retsky et al., 2012). The association between renal cell carcinoma and NSAIDs is inconsistent (Liu et al., 2013).

Harris et al conducted a case control study of NSAIDs among 489 lung cancer patients and 978 control subjects. Matching characteristics included age, gender, and pack-years of cigarette smoking. In order to assess the effects of NSAIDs on tobacco carcinogenesis, only heavy smokers were included in the control group (Harris et al., 2002). The study indicated that daily intake of NSAIDs for at least 2 years prior to interview was associated with a 68% reduction in the relative risk of lung cancer (*p* < 0.01). The inverse trend of lung cancer risk with increasing NSAID use was highly significant (*p* < 0.01). Considering these results with the molecular evidence suggested that regular NSAID intake may prevent tobacco carcinogenesis through COX-2 blockade (Harris et al., 2002).

The present study is also concerned with the effect of NSAIDs in patients with other respiratory diseases. In this study, DNA damage in peripheral lymphocytes of healthy individuals and respiratory disease patients [asthma, chronic obstructive pulmonary disease (COPD), and lung cancer] have been compared before and after treatment with either the bulk (suspension was prepared from the powders as received) and nanoparticle versions of aspirin and ibuprofen in the single cell gel electrophoresis (SCGE) or Comet assay (Tice et al., 2000) and in the micronucleus assay (Fenech, 2007). Also, bulk versions of aspirin and ibuprofen were compared directly with their nano forms. This is the first study to our knowledge that the effects of the particle size of these compounds have been investigated at the cellular level *ex vivo/in vitro*. Our use of lymphocytes is supported by the observations of the WHO/IPCS (Albertini et al., 2000) who reported that lymphocytes are suitable surrogate cells for cancer, and our further work showing that they are suitable surrogates not only for cancer but also for other disease states (Najafzadeh et al., 2012; Anderson et al., 2014), because DNA is the same in all cells of an individual.

The Comet assay was used as a rapid and very sensitive fluorescent microscopic method to assess DNA damage at the individual cell level where broken DNA strands move toward the anode forming a comet shape (Tice et al., 2000; Anderson et al., 2014) and the micronucleus assay (Fenech, 2000) was used for measuring cytogenetic damage. The study of DNA damage at the chromosome level is an essential part of genetic toxicology because chromosomal mutation is an important event in carcinogenesis. The micronucleus assay has emerged as one of the preferred methods for assessing chromosome damage because it enables both chromosome loss and chromosome breakage to be measured reliably. Certain chemicals can be mutagenic and may lead to the induction of micronuclei in cells at the interphase stage. This occurs as a consequence of interference with chromosome structure and/or segregation (Fenech, 2002, 2003, 2007; Kirsch-Volders et al., 2003; Fenech et al., 2009).

There is already another but preliminary communication which was part of this study earlier, responses of lymphocytes after exposure to the bulk compound of ibuprofen and its nanoparticles were investigated by the patch-clamp technique which examines physiological changes in the ion channels of the cell membrane (Shang et al., 2014).

## Materials and methods

### Ethical approval

This present study was approved by Leeds (Central) Research Ethics Committee UK (REC reference number: 09/H1313/37) and the Research Support and Governance Office, Bradford Teaching Hospitals NHS Foundation (ReDA number: 1202). Ethical permission was also provided by the University of Bradford Research Ethics Sub-Committee on Research in Human Subjects (reference number: 0405/8).

The characteristics of patients and healthy individuals are shown in Table 1.

**Table 1:** **Population characteristics**.

**Sample no**.	**Age**	**Gender**	**Ethnicity**	**Smoking history**	**Cigarettes per day**	**Years smoked**	**Pack years**
**LUNG CANCER PATIENTS**							
1	61	M	Caucasian	Past smoker	20	30	30
2^*^	65	M	Caucasian	Smoker	25	40	50
3^*^	68	F	Caucasian	Past smoker	20	>20	20
4^*^	55	M	Caucasian	Smoker	20	30	30
5	63	M	Caucasian	Smoker	20	>40	50
6	65	M	Caucasian	Past smoker	15	>30	23
7	58	M	Caucasian	Smoker	10	30	15
8	63	F	Caucasian	Smoker	40	40	15
9	70	M	Caucasian	No	0	0	0
10	76	F	Caucasian	Past smoker	20	25	25
**CHRONIC OBSTRUCTIVE PULMONARY DISEASE (COPD)**							
11^*^	63	M	Caucasian	Past smoker	Pipe		
12^*^	55	F	Caucasian	Smoker	20	30	30
13^*^	63	F	Caucasian	Smoker	30	30	45
14	61	M	Caucasian	Smoker	20	40	40
15	77	M	Caucasion	Smoker	20	>40	40
16^*^	72	F	Caucasian	Past smoker	20		
17	78	M	Caucasian	Past smoker	20	>40	40
18	54	M	Caucasian	Smoker	5–10	30	11
19	61	M	Asian	Smoker	20	>30	30
20	83	M	Caucasian	No	0	0	0
**ASTHMA**							
21	57	F	Caucasian	No	0	0	0
22	39	F	Caucasian	No	0	0	0
23	38	F	Caucasian	No	0	0	0
24^*^	26	M	Asian	No	0	0	0
25	41	F	Asian	No	0	0	0
26	43	F	CAUCASIAN	NO	0	0	0
27	63	F	Caucasian	No	0	0	0
28	66	F	Caucasian	Past smoker	20	30	30
29	43	M	Asian	Smoker	15	25	10
30^*^	47	M	Caucasian	No	0	0	0
**HEALTHY INDIVIDUALS (NEGATIVE CONTROL)**							
31	68	M	Caucasian	Smoker	5	40	10
32	39	M	Asian	Smoker	5	12	3
33^*^	26	F	Caucasian	No	0	0	0
34	24	M	Caucasian	No	0	0	0
35^*^	40	M	Caucasian	No	0	0	0
36	28	F	Asian	Smoker	5	6	3
37^*^	42	M	Asian	Smoker	10	23	12
38	29	M	Caucasian	No	0	0	0
39	47	F	Asian	No	0	0	0
40	45	M	Caucasian	No	0	0	0

* *Sample numbers with asterisks indicate that individuals who were also examined in the micronuclei assay as well as Comet assay*.

### Whole blood sample collection

The patients were from the Outpatient Respiratory Disease Clinic of Dr. Badie K. Jacobs (St Luke's Hospital, Bradford, UK) and healthy control individuals from the University of Bradford, UK. The criteria for patient selection included lung cancer (without prior chemotherapy or radiotherapy), COPD and asthma. Exclusion criteria were anemia, other diseases beside their respiratory condition or previous occupational exposure to other NPs (e.g., silica or asbestos).

COPD patients were over 40 years of age with a 10 or more pack year smoking history and a fixed spirometric ratio of FEV1 to forced vital capacity (FVC) of <0.7 or radiological evidence of emphysema.

Asthma patients were diagnosed by their medical practitioner. These patients had symptoms of intermittent breathlessness, cough and wheeze. They had less than 10 pack years history of smoking or non-smokers. They had variable and reversible airflow obstruction on spirometery. They also may have had atopy such as allergic rhinitis or hay fever or high IgE with eosinophilia.

All individuals were given an information sheet, completed a questionnaire through interview and signed a consent form prior to approximately 10 ml peripheral blood being taken.

### Non-steroidal anti-inflammatory drugs

Ibuprofen USP was purchased from Albermarle Europe sprl, (Belgium). Pharmcoat 606 (HPMC) and was kindly donated by Shinetsu (Japan). Aspirin and sodium lauryl sulfate (SLS) were purchased from Sigma–Aldrich, UK. Kollidon 30 (PVP K-30) was purchased from BASF (UK). Bulk and nanosuspensions of aspirin and ibuprofen were kindly prepared by Lena nanoceutics (Bradford, UK).

### Nanomaterial methodology

Ibuprofen is a class II drug. This means it is very slightly soluble in water and that the bioavailability of this drug is limited by its dissolution rate. Improving the dissolution rate can therefore ensure the consistency of the drug concentration profile in the blood. Much effort has been directed recently to improve the dissolution rate of class II drugs as their number is predicted to increase. Nanocrystallization is one approach being developed and offers promise. However, there are few data on the pharmacological or toxicological effects of these forms compared to their “bulky” micronized counterpart.

Aspirin is used in the present study to compare its effect to Ibuprofen. Both are NSAIDs. Aspirin, however, is a class III drug and the absorption is the rate limiting step. Nano forms of aspirin could cross the GI tract and therefore improve its bioavailability.

Currently both ibuprofen and aspirin are marketed as micronized particles in tables or suspension. However, there is work on nano forms of these drugs. Companies producing nano forms of ibuprofen could make the claim that their drug has in theory a more consistent bioavailability and plasma drug profile.

Suspensions of aspirin and ibuprofen with solid loads of 3 and 4% (w/w) respectively were prepared. The suspending medium consisted of: HPMC (0.5%, w/w), polyvinylpyrrolidone K-30 (0.5%, w/w) and sodium lauryl sulfate (0.1%, w/w) in deionized water (Plakkot et al., 2011). The milling was carried out using Lena nanoceutics technology DM-100 machine (Sulaiman, 2007).

Two hundred and fifty milliliter of each suspension were milled using 150 ml of 0.2 mm yttrium stabilized zirconium beads (Glen mills, USA). The materials were recycled for 60 min in the milling machine before being discharged in an opaque glass bottle and stored in the refrigerator at (4°C) for the duration of the experiments.

### Zeta potential

The zeta potential for the suspensions was determined using Zetasizer Nano ZS (Malvern Instruments, UK). The suspensions were diluted 1:100 using deionized water and measured at 25°C. Clear disposable zeta cells were used. Measurement duration was set as automatic with a minimum of 10 runs and a maximum of 100 runs. All measurements were made in triplicate.

### Particle size analysis

The particle size distribution of aspirin and ibuprofen nano-suspensions were determined using the dynamic light scattering technique of the Zetasizer Nano ZS (Malvern Instruments, UK). Samples were measured at room temperature using disposable sizing cuvettes. All measurements were carried out in triplicate. The particle size of the stock suspensions were measured in triplicate immediately after milling and then rechecked at the end of the experiments to ensure no significant change in particle size occurred during the various phases of the experiments. The particle size of the bulk powder was measured using the laser diffraction technique (Sympatec Helos, UK) (Ali et al., 2009). Approximately 20 mg of each drug were transferred into the sample vial and the primary pressure was adjusted to 4 bars while the feeder speed was 40 mm/s. Three samples of each drug were measured using R_2_ lens (0.25/0.45, 87.5 μm). Particle size analysis was carried out using dynamic light scattering (zetasizer) (page 10 in the paper under the subtitle “particle size analysis” in the method section. This involved scanning 3 samples, each had a volume of 2–3 ml of the suspension (the concentration of the suspension was 3–4%w/w or 30–40 mg/ml. This provides thousands if not millions of particles to be measured in each sample. The average particle size as well as the poly dispersity index (PDI) are provided in the paper (**Table 3**). Transmission Electron Microscope (TEM) images were there just to show examples of the shape of the particles in the suspension. The particles are suspended in an aqueous solution of a stabilizing polymer and a surfactant. In order to study the sample using XRD it has to be dried which means the polymer will coat the crystals. The amorphous nature of the polymer will create a noisy signal in the XRD diffraction rendering it hard to interpret. This is why no pharmaceutical paper in this area has attempted to measure these samples using XRD. An example of such work includes a paper comparing nano particles produced using wet milling (the same technique in this paper) with micro-fluidic reactors (Ali et al., 2009). The paper focused on the characterization of the suspensions and used varieties of techniques but avoided using FTIR and XRD for the reasons mentioned above. The effect of these additives however on the cells was investigated in our control to eliminate the toxicity effect of these additives. FTIR will suffer from the same drawback as the signal of the water, polymer and the surfactant will create a large noisy background.

Images obtained using TEM. Samples were prepared by placing a drop of the suspension (Aspirin 5% w/w and Ibuprofen 4% w/w) on the TEM carbon grid. The drop was left on the grid for 10 min then the grid was washed by dipping it in water. The grid was floated on a drop of Uranyl acetate for 10 min them washed again with water and left to dry. The grid was then placed in the TEM and the magnification power was 100,000x. The beam intensity was 40,000 KV for Ibuprofen and 60,000 KV for Aspirin sample.

The images confirm that Aspirin crystals are slightly larger than those of Ibuprofen as was shown by the laser diffraction measurements. The images show some smaller particles on the side which might be small crystals of the drugs or it could be aggregates of the polymer used to stabilize the crystals.

### Comet assay

#### Comet assay reagents

Low-melting point agarose, NaCl, EDTA, Tris base, DMSO, Triton X-100, NaOH, EDTA Phythaemagglutinin (PHA) (Cat No. 10576-015) were purchased from Sigma-Aldrich Ltd.

##### The comet assay procedure

Forty blood samples were collected (10 asthma patients, 10 COPD patients, 10 lung cancer patients and 10 healthy individuals from Bradford University).

##### Preparation of whole blood samples

The experiments were carried out on whole blood instead of isolated lymphocytes. The blood sample from each individual was mixed with the same volume of media (RPMI 1640) and 10% DMSO as a cryoprotector and a chelating agent to remove iron effectively. One milliliter of the mixture was aliquot in 1.5 ml Eppendorfs® and transferred to −80°C deep freezer.

##### Cell treatment

Hundred microliter whole blood was incubated in each Eppendorf with five different conditions for 30 min. The first sample was considered as an untreated lymphocyte group without any chemicals and 900 μl RPMI. In the second sample 500 μg/ml of ASP B was added to 100 μl of whole blood and RPMI to the rest of that to make 1000 μl in total. In the third sample, 100 μl of whole blood was added to 500 μg/ml of ASP N and RPMI. The fourth sample, 100 μl of whole blood was added to 500 μg/ml of IBU B and RPMI and finally the fifth sample consisted of 100 μl of whole blood and 500 μg/ml of IBU N and RPMI. The method used thereafter was as described by Tice et al. (2000).

##### Cell scoring

Olive Tail Moment (OTM) was the method used to describe heterogeneity within a cell population and can detect variations in DNA distribution within the tail values; however, % Tail DNA is considered appropriate for regulatory or inter-laboratory comparison studies. Therefore, for all studies that involve multiple electrophoresis runs, it is recommended that % Tail DNA alongside OTM is used to reduce variability in the results (Kumaravel and Jha, 2006; Kumaravel et al., 2009; Azqueta et al., 2014). Fifty cells were scored in duplicate in each patient and healthy individuals.

#### Confounding factors

These were considered for the individuals in this assay using Mann-Whitney test and SPSS version 21 (see later Table 2).

**Table 2:** **Confounding factors**.

**Confounding factors**	***P*-value**
Smoking	*P* < 1.001
Age	*P* < 0.437
Ethnicity	*P* < 1.000
Drinking habit	*P* < 0.203
Gender	*P* < 0.20

#### The cytokinesis block micronucleus (CBMN) assay

General laboratory reagents were purchased from Fisher Scientific Co. (Itasca, IL, USA). RPMI 1640 medium (with L-glutamine and 25 mM Hepes), Cytochalasin B (Cat No. C6762), and mitomycin C (Cat No. M0503) were purchased from Sigma-Aldrich (Poole, UK), penicillin-streptomycin solution (Cat No.15140-122) were purchased from Invitrogen Ltd, UK. Slides and coverslips were obtained from VWR international. The Fixogum rubber cement was purchased from (Marabu, Germany).

##### Micronucleus procedure

Six different blood samples used in this study consisted of: 5 healthy control individual, 1 asthma patient, 1 COPD patient and 3 lung cancer patients.

Under sterile conditions, culture medium was prepared. Streptomycin solutions were added to RPMI -1640 medium containing 25 mM HEPES and L-Glutamine, with end concentrations of 15 and 1%, respectively. Hundred milliliter of this medium were aliquoted to 4.5 ml into T 25 cm2 Corning culture flasks and stored at −20°C. The T25 cm^2^ flasks were equilibrated in a 37°C incubator (5% CO_2_) prior to cell culture use.

##### Cell treatment

Start of culture: 500 μl of blood was added to a T25 cm^2^ culture flask containing 4.5 ml medium, followed by 100 μl of phythaemagglutinin (PHA). In the next 24 h, 125 μl of test substances were added in each flask followed by 750 μl of H_2_O, to give an end concentration of 125 μg/ml. For the negative control, 125 μl of standard control solution was used. Cultures were incubated at 37°C in the presence of 5% CO2 for 44 h.

##### Cell scoring

Using bright-field light microscopy at 40 × magnification, slides were scored using criteria, as recommended by Fenech et al. (2003), Fenech (2007). Micronuclei (MNi) were scored each from binucleated (BiNC) and mononucleated (MonoNC) cells. The nuclear division index (NDI) was used as an indicator of the cytotoxicity and the following calculation was used to find the NDI: NDI = (M1 + 2(M2) + 3 (M3) /N.

Where: M1 = mononucleated cells, M2 = binucleated cells, M3 = multinucleated cells, N = the total number of viable cells scored (Fenech, 2007).

##### Statistical tests for the comet assay

Gaussian normality was violated for many of the scale variables as endorsed by the Kolmogorov–Smirnov test. Therefore, non-parametric test procedures were adopted wherever necessary, such as the Kruskal–Wallis (K–W) and the Mann–Whitney (M–W) tests for independent samples. When testing intra-subject differences in DNA damage, the Wilcoxon Signed Rank test was applied (Najafzadeh et al., 2012).

##### Statistical analysis for the micronucleus assay

This was carried out using the chi-square test (X^2^) in 1000 cells per group to determine significant differences with *p*-value set at ^*^p ≤ 0.05 as recommended by Fenech (2003).

## Results

### Dose ranging study in lymphocytes

Three different doses each of both the bulk and nanosuspensions, of aspirin and ibuprofen, were used to examine genotoxic damage with the Comet and micronucleus assays in blood samples. These doses were 250, 500, 1000 μg/ml. In this study it was determined that 500 μg/ml in the Comet assay and 250 μg/ml in the micronucleus assay were optimal doses with no cellular toxicity for the two suspensions of aspirin and ibuprofen.

### Solubility

Aspirin and ibuprofen are two NSAIDs that have different levels of solubility. Aspirin is reported to have a solubility value of 10 mg/ml and ibuprofen has a solubility value of 21 mg/ml. The concentration used in this experiment was 500 μg/ml which is below the solubility value of aspirin and higher than that of ibuprofen. The nanosuspension of aspirin was less stable than that of ibubrofen and was made up freshly every few weeks.

### Excipient response

The effect of the excipients in the suspensions (bulk suspensions) on the cells was investigated by treating with a solution containing the same concentration of the excipient without ibuprofen and aspirin. The results showed no significant difference between the excipient and the untreated cells.

For the Comet and micronucleus assays in all four groups (healthy individuals, asthma, COPD and lung cancer) bulk forms were compared directly to their nano forms. Also all treated samples in each group were compared to their untreated lymphocytes.

#### Particle size analysis of aspirin and ibuprofen and TEM images

**Figure 1:**
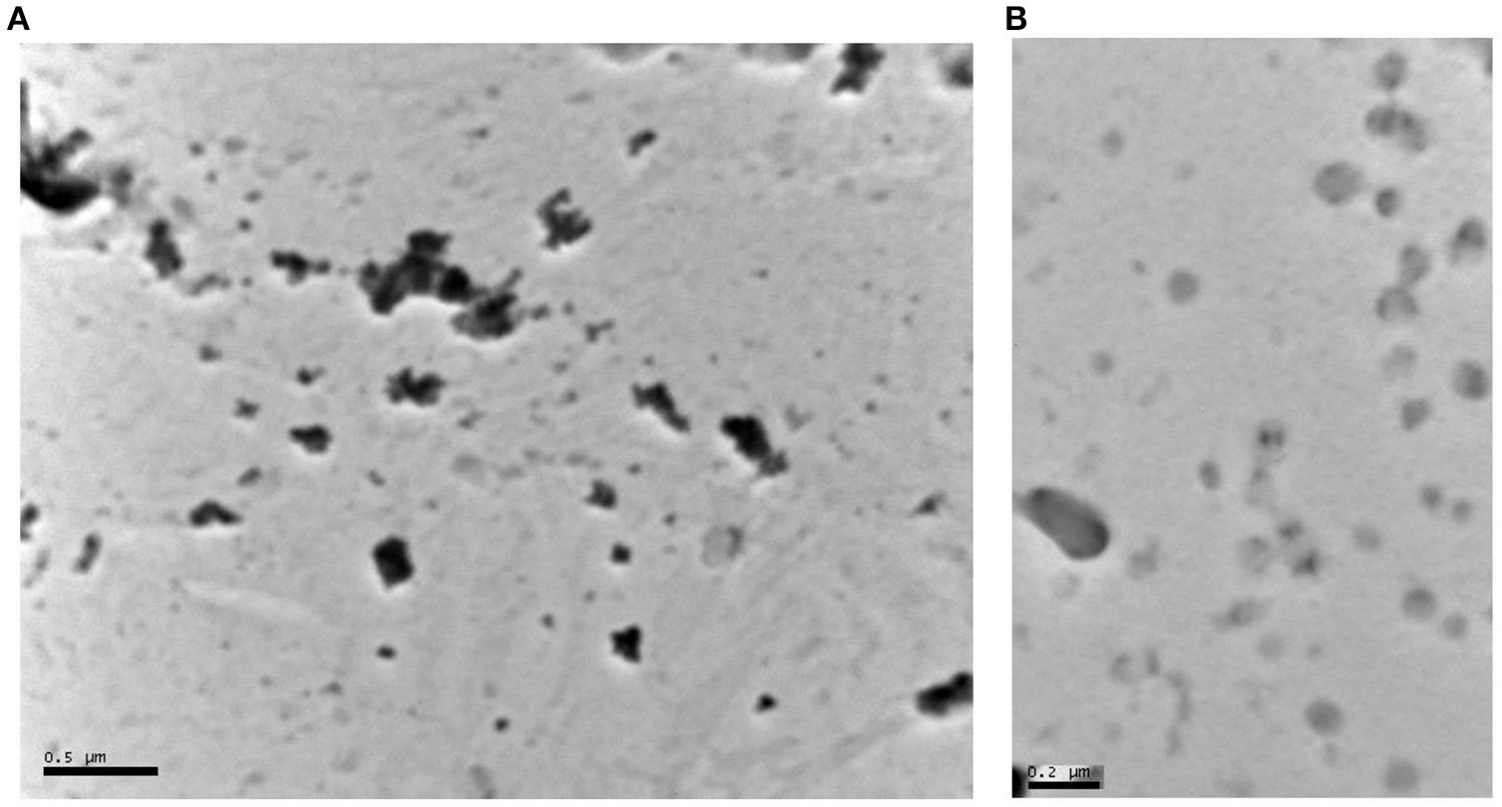
**(A)** Transmission Electron Microscope (TEM) image of aspirin nanoparticles precipitated on a carbon grid. The dark particles in the image range in size between 50 and 200 nm. The particles appear as individual particles or clusters of small particles. The black dots are the crystals of aspirin (separate or in clusters). The gray dots are particles that are not in focus in the image or polymer aggregates (the stabilizing excipients: polyvinylpyrrolidone K-30 and hydroxylpropyl methylcellulose). **(B)** Transmission Electron Microscope image of Ibuprofen nanoparticles precipitated on a carbon grid. Ibuprofen particles are less dense and therefore appear less dark than aspirin in the image range in size between 30 and 200 nm. The particles appear as individual particles or clusters of small particles. Ibuprofen particles are gray as they are more transparent to the electron beam. The particles that are not in focus are either more transparent or blending in the background.

The average particle size of the nanosuspensions, their poly dispersity *indices* (Pdi) and their Zeta potential are reported in Table 3. Moreover, the average particle size and the volume mean diameter of the bulk powder are shown in Table 4. The TEM images of aspirin and ibuprofen are shown in Figures 1A,B.

**Table 3:** **Average particle size, polydispersity index, and zeta potential values of the nano-suspensions (***n*** = 3)**.

**Suspension name**	**Time of measurement**	**Average particle size (nm)**	**Polydispersity index**	**Zeta potential value (mV)**
Ibuprofen nano-suspension 3%	Before cell treatment	323 ± 6.4	0.2 ± 0.01	−2.1
	After cell treatment	340 ± 1.2	0.3 ± 0.001	
Apsirin nano-suspension 4%	Before cell treatment	289 ± 3	0.3 ± 0.03	−6.1
	After cell treatment	299 ± 6.3	0.3 ± 0.05	

**Table 4:** **Average particle size (x_**90**_) and the volume mean diameter of the bulk powder (as received) of aspirin and ibuprofen (***n*** = 3)**.

**Suspension name**	**Average particle size (μm)**	**Volume mean diameter (μm)**
Ibuprofen	52.80 ± 4.37	20.50
Apsirin	78.30 ± 0.23	44.57

#### Comet assay

**Figure 2:**
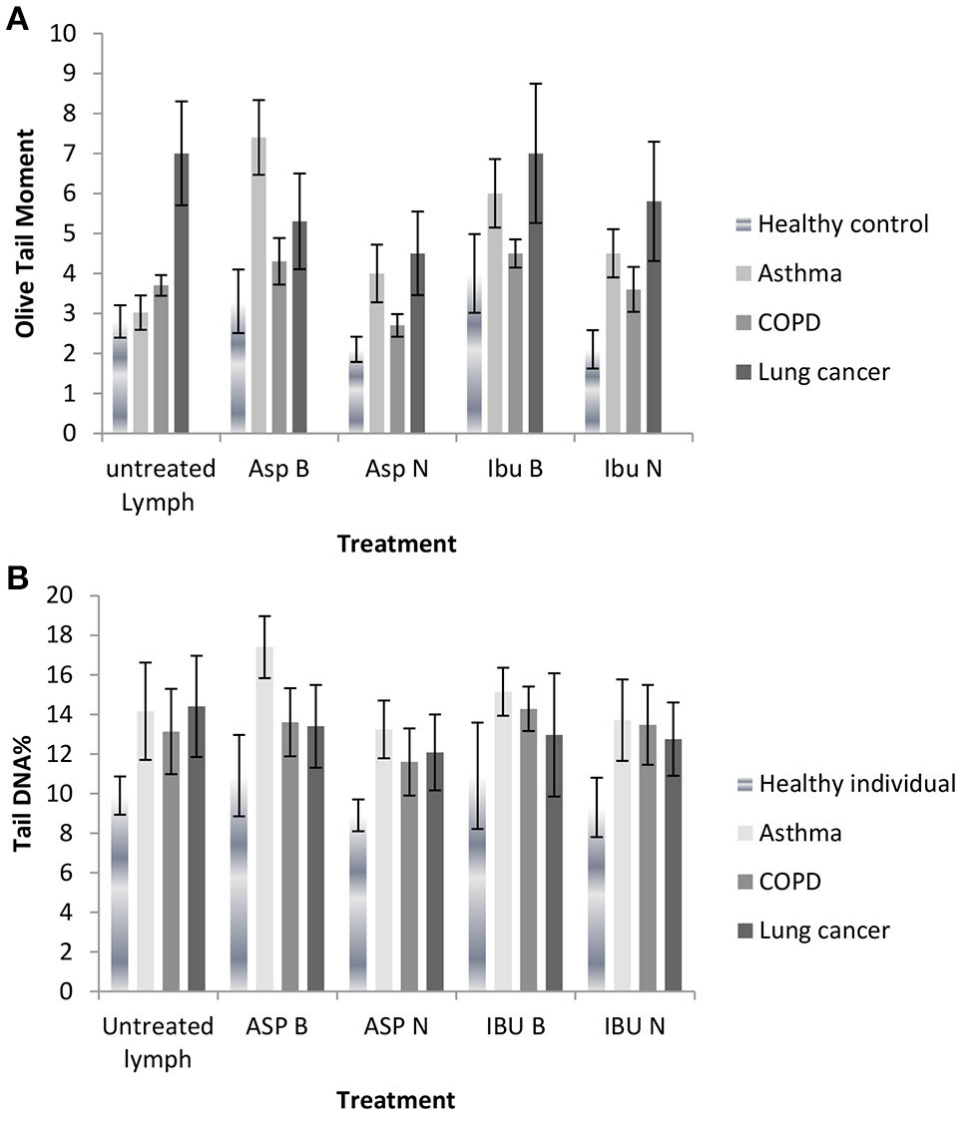
**(A)** DNA damage measured as Olive tail moments before and after treatment with nano and bulk forms of aspirin and ibuprofen in lymphocytes from healthy individuals and patient groups in the Comet assay. **(B)** DNA damage measured as % tail DNA before and after treatment with nano and bulk forms of aspirin and ibuprofen in lymphocytes from healthy individuals and patient groups in the Comet assay.

As shown in Figures 2A,B, and Tables 5A,B there were four different groups of treatment [aspirin bulk (ASP B), aspirin nano (ASP N), ibuprofen bulk (IBU B) and ibuprofen (IBU N) and also an untreated lymphocyte group] in each experiment.

**Table 5:** **DNA damage from nano and bulk forms of aspirin and ibuprofen in the lymphocytes from patient groups and healthy individuals compared to untreated lymphocytes in the Comet assay (Olive Tail moments, A) (%Tail DNA, B)**.

**(A)**					
**Study groups**	**Untreated lymphocytes OTM**	**ASP B OTM**	**ASP N OTM**	**IBU B OTM**	**IBU N OTM**
Healthy individuals	2.83 ± 0.40	3.33 ± 0.80^¥¥^	2.1 ± 0.3^**^^‡‡^	3.97 ± 1.00	2.08 ± 0.50^**^
Asthma	3.03 ± 0.43	7.42 ± 0.93^¥¥^	4.02 ± 0.72^**^	6.21 ± 0.86	4.49 ± 0.60^**^
COPD	3.76 ± 0.26	4.32 ± 0.60^¥¥^	2.69 ± 0.28^‡‡^	4.53 ± 0.35	3.61 ± 0.56^**^
Lung cancer	7.00 ± 1.30	5.37 ± 1.20^§§^	4.55 ± 1.05^**^^‡‡‡^	6.98 ± 1.74	5.78 ± 1.50^**^^‡‡^
**(B)**					
**Study groups**	**Untreated lymphocytes %Tail DNA**	**ASP B %Tail DNA**	**ASP N %Tail DNA**	**IBU B %Tail DNA**	**IBU N %Tail DNA**
Healthy individuals	9.9 ± 0.96	10.9 ± 2.05^¥¥^	8.9 ± 0.85^**^	10.9 ± 2.69	9.3 ± 1.50^**^
Asthma	14.16 ± 2.05	17.4 ± 1.41^¥¥^	13.24 ± 1.01^**^	15.14 ± 1.23	13.7 ± 1.97^**^
COPD	13.13 ± 2.15	13.6 ± 1.72^¥¥^	11.6 ± 1.70^**^	14.28 ± 1.13	13.47 ± 2.01^**^
Lung cancer	14.4 ± 2.55	13.4 ± 2.09^§§^	12.07 ± 1.91^**^^‡‡‡^	12.96 ± 3.11	12.75 ± 1.86^**^^‡‡^

Decrease in significance:

Nano suspension

** *P < 0.01 nano suspension compared to bulk suspension*.

‡‡ *P < 0.01 ASP N suspension compared to untreated lymphocytes*.

‡‡‡ *P < 0.01 IBU N suspension compared to untreated lymphocytes*.

Increase in significance:

Bulk suspension

¥¥ P < 0.01 Bulk suspension compared to untreated lymphocytes except for lung cancer

§§ *P < 0.01 Bulk suspension compared to untreated lymphocytes*.

DNA damage decreased in lymphocytes from healthy individuals, asthma, COPD and lung cancer patient groups after treatment with aspirin nanosuspension (ASP N) and ibuprofen nanosuspension (IBU N) compared to their bulk version (micro-suspension) in the Comet assay (*P* ≤ 0.01). However, when ASP N was compared to untreated lymphocytes in all groups in the Comet assay, DNA damage significantly decreased in all groups, except the asthma group. When IBU N was compared to untreated lymphocytes, in healthy individuals and the lung cancer group, DNA damage decreased, but increased in asthma and COPD groups.

DNA damage in lymphocytes from healthy individuals, asthma and COPD in the untreated lymphocyte group compared to the cells treated with ASP B and IBU B was significantly increased both for OTM and % tail DNA (*P* ≤ 0.01) except for the lung cancer group.

In lung cancer patients after treating lymphocytes with ASP B and IBU B the DNA damage was decreased significantly (*P* ≤ 0.01) and further reduction after treatment with ASP N (*p* ≤ 0.001) and IBU N (*P* ≤ 0.01) was noted.

#### Confounding factors in the comet assay

The confounding factors such as gender, smoking, drinking habit, ethnicity and age were considered, and there were no significant differences between the results in relation to these parameters (Table 2).

#### Binucleated cells (BiNC)

For each treated group (healthy individuals, asthma, COPD, and lung cancer (Table 6), there was generally a decrease in the number of MNi in binucleated cells after treatment in healthy individuals and lung cancer groups when compared to the untreated lymphocytes. There was also a reduction in the number of MNi with ASP N compared to the ASP B, and there was a similar reduction in the number of MNi with IBU N compared to IBU B between different treatment types, per group.

**Table 6:** **The effect of nano and bulk forms of aspirin and ibuprofen on the lymphocytes from patient groups and healthy individuals compared to untreated lymphocytes in the micronucleus assay**.

**Subject**	**Treatment**	**% BiNC**	**% Multi**	**MNi in BiNC cells**			**Mono**
				**BiMNi**	**BiNPB**	**BiBuds**	**MNi**
Healthy individuals	Untreated lymphocytes	50.025 ± 2.02	28.52 ± 0.56	6.00 ± 1.02	3 ± 0.02	0	0
	Positive control	59.12 ± 4.07	13.49 ± 0.44	47.75 ± 0.25	1.75 ± 0.77	1.25 ± 0.5	11.75 ± 1.32
	Aspirin—B	53.775 ± 2.03	24.83 ± 1.52	5.22 ± 0.16	1.25 ± 0.04	0	3.5 ± 0.02
	Aspirin—N	59.55 ± 0.06	13.55 ± 1.09	4 ± 0.03	0.25 ± 0.02	0	1 ± 0.45
	Ibuprofen—B	57.8 ± 1.86	15.9 ± 0.09	5.75 ± 0.14	1 ± 0.125	0	2 ± 0.02
	Ibuprofen – N	54.91 ± 2.09	31.56 ± 1.62	4.5 ± 0.06	1 ± 0.4	0	1.5 ± 0.3
Asthma	Untreated lymphocytes	42.04	36.16	4	0	0	0
	Positive control	44.17	39.16	19	0	0	4
	Aspirin—B	42.44	29.05	5	1	1	1
	Aspirin—N	50.1	16.11	2	1	0	4
	Ibuprofen—B	47.5	25.14	8	0	2	0
	Ibuprofen—N	52.13	28.2	6	0	0	4
COPD	Untreated lymphocytes	57.42	2	10	0	0	1
	Positive control	25.4	0.3	17	0	2	1
	Aspirin—B	48.5	0.5	13	0	0	4
	Aspirin—N	45.22	0.3	10	0	0	1
	Ibuprofen—B	41.43	0.2	14	0	0	9
	Ibuprofen—N	45.02	0.1	16	0	0	1
Lung cancer	Untreated lymphocytes	46.33 ± 4.40	35.34 ± 0.48	22.67 ± 2.06	5.21 ± 0.22	3.33 ± 1.24	14.66 ± 4.01
	Positive control	44.66 ± 0.06	35.66 ± 0.02	32.33 ± 0.34	2.23 ± 0.12	5.33 ± 0.04	2.00 ± 0.01
	Aspirin—B	43.33 ± 0.07	29.33 ± 0.67	15.66 ± 2.77	3.33 ± 0.08	4.66 ± 0.26	3.00 ± 0.03
	Aspirin—N	45.00 ± 0.12	29.66 ± 0.27	7.66 ± 0.02	0	2.33 ± 0.33	2.6 ± 0.63
	Ibuprofen—B	42.33 ± 0.34	27.33 ± 0.05	11.00 ± 0.03	2.33 ± 0.01	3.66 ± 0.04	9.33 ± 0.15
	Ibuprofen—N	47.33 ± 0.01	29.00 ± 0.12	9.00 ± 0.14	1.33 ± 0.56	0	2.66 ± 0.05

*BiNC, Binucleated cells, % BiNC, is % expressed out of all types of 1000 (500 × 2) cells scored; MonoNC, Mononucleated cells; MultiNC, Multinucleated cells % MultiNC, is % expressed out of all types of 500 cells scored; MNi, Micronuclei score/1000 (500 × 2) cells each of BiNC and MonoNC*.

#### Mononucleated cells (MonoNC)

These are cells which have not undergone cell division. For each group, the number of MNi was generally higher in BiNC cells compared to Mono NC cells.

There was a decrease in number of MNi in cells treated with both ASP B and IBU B when compared to their untreated lymphocytes in cancer patients and healthy individual. Comparing the bulk and the nano forms, there was a reduction in the number of micronuclei treated with ASP N and IBU N when compared to their bulk forms. Overall, the data of binucleated cells and mononucleated cells indicate that there is a reduction in DNA damage in blood samples treated with nano forms of aspirin and ibuprofen over their bulk counterparts. DNA damage decreased in lymphocytes from healthy individuals, asthma, COPD and lung cancer patient groups after treatment with ASP N and IBU N compared to their bulk version (micro-suspension) in both assays. However, when ASP N and IBU N were compared to untreated lymphocytes in healthy individuals and the lung cancer group in both assays, DNA damage significantly decreased, but this was not the case for micronucleus frequency in asthma and COPD patients.

#### Ibuprofen treatment in nanoparticle and bulk suspensions on ion-channel activities of lymphocytes

With the patch-clamp technique, whole-cell current recordings from ibuprofen -treated lymphocytes were performed. A representative whole-cell recording obtained from the treated lymphocytes with 500 μg/ml of different treatments is presented. When compared to untreated cells, lymphocytes treated with IBU N had lower whole-cell currents. The current–voltage (I–V) relationships for these recordings are shown. In the presence of nanoparticles, the amplitude of the total current (pA/pF) was decreased at the test potentials between −50 and +0 mV. These whole-cell currents were consistently evoked by applying 10 mV steps of 1000 ms duration from a hold potential of −50 to +30 mV.

## Discussion

With regard to the solubility of aspirin and ibuprofen, it is well documented that the dissolution rate and extent from nanoparticles are many times higher than that of micronized particles. Due to the large difference in surface area between micronized and nanoparticles the diffusion of aspirin will be different, not to mention the possibility of nanoparticles crossing the cell membrane before dissolution. Our results below prove that there is a difference in response between the micronized and nano form of aspirin. In all cases the comparison between the two NSAIDs was the one of the aims of this project and the results supported our expectation to the differences between the two drugs.

Briefly, the present study has examined DNA damage in the Comet and micronucleus assays in peripheral blood lymphocytes of patients with respiratory diseases and healthy individuals using the nanoparticle (NP) and bulk suspensions of the NSAIDs, aspirin and ibuprofen. DNA damage decreased in lymphocytes from healthy individuals, asthma, COPD and lung cancer patient groups after treatment with ASP N and IBU N compared to their bulk version in both assays. However, when ASP N was compared to untreated lymphocytes in all groups in the Comet assay, DNA damage significantly decreased in all groups, except the asthma group. When IBU N was compared to untreated lymphocytes, in healthy individuals and the lung cancer group, DNA damage decreased, but increased in the asthma and COPD groups. Similarly, when comparing the bulk and nanosuspensions, DNA damage in the micronucleus assay followed the same pattern as for the Comet assay, where the nano version produced less micronuclei (MNi) than the bulk. Frequency of micronuclei (MNi) decreased after ASP N and IBU N in the healthy individual and lung cancer groups, and also decreased in COPD and asthma groups except for IBU N. Furthermore, lymphocyte responses after IBU N and IBU B were investigated by the physiological patch-clamp technique. The patch-clamp experiments where only IBU bulk and nano forms have been investigated demonstrated that IBU N inhibited ion channel activity by 20%. This study also compared bulk and nano versions and showed that nano form produced a more marked response (Shang et al., 2014).

Based on previous studies there is no chemopreventive agent that has been specifically designed for lung cancer, though there are several promising areas to investigate (Greenberg et al., 2013). Also there are a large number of clinical and preclinical studies that have presented strong data to show NSAIDs could inhibit various types of cancers as described earlier (Kawahara et al., 2010; Thapa et al., 2010; Brady et al., 2011; Coghill et al., 2011, 2012; Retsky et al., 2012; Kang et al., 2013; Seufert et al., 2013). NSAIDs act as inhibitors of the cyclooxygenase (COX) enzymes which convert arachidonic acid to prostaglandin E2. The COX enzymes play an important role in inflammation, and a large number of COX-2 dependent genes are involved in tumorigenesis (Kim et al., 2010; Xu et al., 2012). COX-2 is often up-regulated in carcinoma-*in-situ* and non-small cell lung cancer (Krysan et al., 2006). In a recent study, Naproxen which is a strong NSAID was examined to find the effects on urinary bladder cancer cells in human and rat bladder cancers. Naproxen made an accumulation of cells at the G1 phase mediated through CDK4, cyclin D1 and p21 and a significant apoptosis. The apoptosis largely was due to down-regulation of Bcl-2 and up-regulation of Bax was involved (Kim et al., 2014).

Since cancer nano therapeutics has progressed in the field of research and development in the last decade, there is significant anticipation that nanoparticle technologies may the key to designing successful cancer treatment (Bertrand et al., 2014).

Inflammation is one of the critical factors in carcinogenesis, and many investigators have focused on anti-inflammatory agents, for instance glucocorticoids, NSAIDs, statins, and PPARγ agonists. Although, successful chemoprevention requires subjecting the multiple pathways to carcinogenesis with the lowest toxicity and highest efficacy (Greenberg et al., 2013).

This molecular epidemiology approach mirrors pre-clinical and clinical findings, with regards to NSAIDs it also provides information on bulk vs. nano-forms of these compounds and also shows how disease state individuals might differ in their response.

## Author contributions

DA and MN conceived and designed the experiments. MN performed the Comet and Micronucleus assays. CN helped with the Micronucleus assay. BJ provided the blood samples. MI and RG were involved with NP preparation. Data were analyzed by MN. MN and DA co-wrote the paper.

## Funding

The present study was part funded by United Kingdom India Education Research Initiative (UKERI) SA 07-067.

### Conflict of interest statement

The authors declare that the research was conducted in the absence of any commercial or financial relationships that could be construed as a potential conflict of interest.
